# Does the perception of fairness and standard of care in the health system depend on the field of study? Results of an empirical analysis

**DOI:** 10.1186/1472-6963-14-166

**Published:** 2014-04-12

**Authors:** Kathrin Damm, Anne Prenzler, Andy Zuchandke

**Affiliations:** 1 Leibniz University Hannover, Center for Health Economics Research Hannover (CHERH), Königsworther Platz 1, D-30167 Hannover, Germany; 2 Leibniz University Hannover, Center for Risk and Insurance, Königsworther Platz 1, D-30167 Hannover, Germany

**Keywords:** Fairness, Standard of care, Health care, Perception, Field of study, Germany

## Abstract

**Background:**

The main challenge in the context of health care reforms and priority setting is the establishment and/or maintenance of fairness and standard of care. For the political process and interdisciplinary discussion, the subjective perception of the health care system might even be as important as potential objective criteria. Of special interest are the perceptions of academic disciplines, whose representatives act as decision makers in the health care sector. The aim of this study is to explore and compare the subjective perception of fairness and standard of care in the German health care system among students of medicine, law, economics, philosophy, and religion.

**Methods:**

Between October 2011 and January 2012, we asked freshmen and advanced students of the fields mentioned above to participate in a paper and pencil survey. Prior to this, we formulated hypotheses. The data were analysed by micro econometric regression techniques.

**Results:**

Data from 1,088 students were included in the study. Medical students, freshmen, and advanced students perceive the standard of care significantly as being better than non-medical students. Differences in the perception of fairness are not significant between the freshmen of the academic disciplines; however, they increase with the number of study terms. Besides the field of study, further variables such as gender and health status have a significant impact on perceptions.

**Conclusions:**

Our results show that there are differences in the perception of fairness and standard of care between academic disciplines, which might influence the interdisciplinary discussion on health care reforms and priority setting.

## Background

The problem of scarce resources leads to debates about financing and service provision in health care systems. A main challenge in this context is the establishment and/or maintenance of fairness and standard of care.

Using objective assessment criteria, Germany seems to have a high standard of care (e. g. in terms of right, safe and patient-centered care) and a fair system in comparison to other countries [1-3]. Health care provision is independent of income and social status, and co-payments are relatively low and limited [4]. Further benchmark criteria, as e.g. provided by Norman Daniels are also met (relatively high patient autonomy and equality in coverage and quality of care) [5,6].

The judgment whether a system is good and/or fair can nevertheless depend highly on the subjective perception of individuals. For the political process as well as important interdisciplinary discussions on health care reforms, priority setting and rationing, subjective perceptions of the health care system might even be as important as potential objective criteria. In this context, the perceptions from the academic disciplines that are involved in the discourses are of special interest since their representatives act as decision makers in the health care sector. These include physicians, lawyers, philosophers of ethics, economists, and religious figures.

In particular the interdisciplinary discussion on priority setting (making a relative ranking of health care programmes, services or types of patients) and rationing (distributing scarce resources when no market exists) between these academic disciplines is quite challenging, due to the different methodologies and approaches used in each discipline [7-9]. One controversial approach, for example, is that of the incremental cost-effectiveness ratio (ICER), offering transparency with respect to the additional costs and benefits of new health technologies in comparison with those of standard interventions, and being an important outcome in health economic evaluations (e.g. in cost-utility analyses [CUA]) [10]. However, with exceptions, [11] prominent researchers with philosophical or legal backgrounds have argued that these kind of analyses (as instruments of utilitarian benefit maximization) led to discrimination against individuals (older or sicker patients, expensive or rare indications) and counteracts the principle of equal chances of and access to health care [12-14]. They indicate that consequentialist approaches - unlike approaches of procedural justice - cannot fulfil the principle of equality between citizens or argue for an general moral right to health care [15]. Giving priority to the worse-off or those with the highest needs is a principle enrooted in medicine but empirical studies of physicians' behaviour also identified tendencies of rationing by age [16-18].

The different methodologies and approaches used and taught in academic disciplines may result in systematically different opinions on the problem of scarce resources and possible solutions, [19,20] which in turn gives rise to the hypothesis that educational background may influence perceptions of the health care system. Advanced medical students, for example, might assess the standard of health care differently than non-medical students, while philosophy students might perceive the health care system to be less fair due to a deep insight into the theory of justice. In this context, it is interesting to investigate whether perception changes during the years of education or is already determined before the students take up their studies in a specific subject at the university.

The aim of this study is to explore and compare the subjective perception of fairness and standard of care in the German health care system among students of medicine, law, economics, philosophy, and religion, freshmen as well as advanced students since systematic differences might give another explanation for challenging interdisciplinary discussions on health care financing and service provision. For this, we developed and tested a unique questionnaire. We also formulated hypotheses and used micro econometric regression techniques to explore them.

### Hypotheses

*Hypothesis 1*: *Incoming students perceptions towards standards of care and fairness do not systematically differ between different fields of study*.

In general, most students who continue to study at the university level do so immediately after receiving their high school diploma. A relatively uniform educational background can therefore be assumed. Solely the medical freshmen might have a particular opinion with respect to standard of care. Although Zupanic et al. concluded that the main motivations to study medicine are an interest in medical issues, contact with people, and the desire to help, the results give no indication about a specific perception toward standard of care [21]. Accordingly, we assume that the freshmen`s perception does not differ systematically between the different fields of study.

*Hypothesis 2*: *Advanced medical students assess the standard of care as being worse compared to the assessments of incoming medical students. In addition*, *advanced medical students view the standard of care as being worse compared to the assessments of advanced students in any other field of study*.

Unlike non-medical students, medical students learn about the capabilities of medical care. At the same time, studies show that German medical students anticipate the budgeting of services as an important problem of their future work. They perceive deficiencies in patient care and are concerned about them in the future [21,22]. Additionally, Griffith and Wilson report a loss of idealism during clinical rotations [23]. Since non-medical students do not deal with standard of care in the German health care system in detail, we do not assume a change in their perception.

*Hypothesis 3*: *Advanced philosophy*/*religion students assess the fairness of the German health care system as being worse compared to the assessments of incoming philosophy*/*religion students. Furthermore*, *advanced philosophy*/*religion students view the fairness of the German health care system as being worse compared to the assessments of advanced students in any other field of study*.

While medical students potentially develop a more negative perception with respect to standard of care, advanced students of philosophy or religion might assess the fairness in the system as worse. A reason for this might be that students of philosophy or religion learn to examine issues and theories of justice more critically during their studies [24,25]. Regarding this some initial evidence was found by Annis and Annis [26]. Accordingly, we assume more critical responses here.

## Methods

To test our hypotheses empirically, we asked students of law, economics, philosophy, and religion at the University of Hannover (LUH) as well as medical students at the Hannover Medical School (MHH) to fill out a questionnaire that was specifically designed for this study. To enlarge the group of philosophy and religion students, we also included students of the University of Göttingen. While the pre-test was carried out in September 2011, the main study was conducted between October 2011 and January 2012. Ethical approval for this study was given by the Ethics Committee of the MHH. Students were informed about the aim of the study, the optional nature of participation as well as the anonymity of their responses. A written informed consent was not needed.

We decided to use the following statement as a proxy for health care standard:

‘*If a person becomes seriously ill in Germany*, *the system provides very good health care*’. (*in German*: *Wenn man in Deutschland ernsthaft krank wird*, *wird man sehr gut versorgt*).

To explore the perception of fairness in the German health care system, we integrated the following statement:

‘*I think that our health care system is fair*’. (*in German*: *Ich finde*, *dass unser Gesundheitssystem gerecht ist*).

Fixed choice responses were given in a 5-point Likert scale, from ‘strongly disagree’ to ‘strongly agree’, with the middle category ‘neutral’. The questionnaire also included information on the academic discipline, number of study terms (in Germany, a term or ‘Semester’ lasts half the academic year), health status, and socio-economic background of the respondents. Freshmen were questioned mainly during their ‘study orientation week’ at the beginning of their first study term. We also collected data from advanced students, since we assume that the length of the study has an influence on perception. Questionnaires were filled out anonymously in lectures or breaks. Data entry, cleanup, and analysis were performed with the software SPSS (version 19) and Stata 11.

To test our hypotheses within the regressions, we use the nominal-scaled variable ‘academic discipline’. Due to the small number of students studying philosophy and religion as well as the fact that most students took classes in both subjects (as major and minor), we assigned the responses of these students to one group. The possible answers ‘medicine’, ‘law’, ‘economics’, and ‘philosophy/religion’ were converted to dummy variables. We also included the variable ‘study terms’, which refers to the number of terms that the students had already completed. Freshmen therefore have ‘zero’ study terms. Finally, we created interaction variables (study terms × academic discipline) to identify the effect of the variable ‘study terms’ for all academic disciplines separately.

We included a set of control variables in our regressions. Besides gender, nationality (German or other), and whether respondents have children, additional binary variables indicate the existence or non-existence of an acute or chronic disease as well as the presence of a serious illness among friends or family members. In addition, the type of health insurance indicates whether the respondents are insured by statutory health insurance (SHI) or private health insurance (PHI). In Germany, most students are covered via a parent or alternatively pay a reduced contribution rate, so that there is hardly any difference from a financial point of view. There are nevertheless substantial differences in the benefits catalogue. While PHI reimburses nearly all available medical products and services, the SHI catalogue is restricted. In addition, the remuneration of physicians in the outpatient and inpatient setting differs between both types of insurances. Due to higher reimbursement, privately insured persons get appointments earlier and receive more comprehensive treatment than those covered by SHI [27-29]. In addition, we included three more control variables regarding the students’ individual health status, health conscious living, and social commitment, all requested by a 5-point likert-scale. For the analysis, we coded these variables in three categories. As a proxy for the social background of the respondents, we used parental education. The highest parental educational level, which we had originally requested in the questionnaire, was recoded into the number of typically completed schooling or training years. Finally, we included the age.

Further information on all control variables is provided in Table 1.

**Table 1:** Variables included in the regressions

**Variable**	**Average (standard error)**
HYPOTHESES RELATED	
Medicine (*0* = *no 1* = *yes*)	0.29 (0.452)
Law (*0* = *no 1* = *yes*)	0.33 (0.472)
Economics (*0* = *no 1* = *yes*)	0.29 (0.453)
Philosophy/religion (*0* = *no 1* = *yes*)	0.09 (0.289)
(Finished) study terms^1^ (*number*)	1.64 (2.576)
FURTHER CONTROL VARIABLES	
Gender (*0* = *male 1* = *female*)	0.60 (0.489)
Nationality (*0* = *German 1* = *other*)	0.05 (0.214)
Children (*0* = *no 1* = *yes*)	0.03 (0.161)
Type of health insurance (*0* = *SHI 1* = *PHI*)	0.18 (0.387)
Acute disease (*0* = *no 1* = *yes*)	0.09 (0.279)
Chronic disease (*0* = *no 1* = *yes*)	0.14 (0.347)
Serious illness among family or friends (*0* = *no 1* = *yes*)	0.35 (0.475)
Subj. health status (-*1* = *bad*–*1* = *good*)	0.84 (0.462)
Subj. health consciousness ((-*1* = *no*–*1* = *yes*)	0.59 (0.686)
Social commitment (-*1* = *no*–*1* = *yes*)	-0.20 (0.906)
Parental schooling years (*number*)	14.72 (4.042)
Age	22.47 (2.749)

^1^Starting with 0 for the first study term.

SHI: statutory health insurance, Subj.: Subjective, PHI: private health insurance.

To evaluate our hypotheses, we consider the following initial equation for our estimation approach:

(1) $$\begin{array}{l} y_{i}^{*}=\beta_{0}+\beta_{1}\cdot \mathrm{ter}m_{i}+\gamma^{\prime }\cdot M_{i}+\varphi^{\prime }\cdot \mathrm{ter}m_{i}\cdot M_{i}\\ \;+\mu^{\prime }\cdot X_{i}+\epsilon_{i} \end{array}$$

where $y_{i}^{*}$ represents the non-observable continuous variable on the perception of either fairness or standard of care. The variable ‘study terms’ indicates the number of terms and the vector *M*_*i*_ includes the dummy variables for the different disciplines philosophy/religion, law, economics, and medicine. In the regression analysis regarding the standard of care, the variable ‘medicine’ is considered as the basis due to the formulation of hypothesis 2. In the analysis with respect to fairness (hypothesis 3), we use ‘philosophy/religion’ as the basis variable. The vector *X*_*i*_ includes all control variables. Furthermore, *ϵ*_*i*_ is the idiosyncratic error term, which we assume to be normally distributed. As can also be seen in Equation 1, the product of the variable *term*_*i*_ and the vector *M*_*i*_ represents the interaction terms mentioned above. As a consequence of including the interaction terms, the coefficient *β*_1_ represents only the effect of terms of the respective basis variable (medicine when considering standard of care and philosophy/religion when considering fairness). The influences of the other disciplines are covered by the interaction effects.

As we only observe an ordinal structure of two dependent variables, we make use of an ordered probit regression based on a latent variable approach. The probit specification is used due to our assumption of the normally distributed idiosyncratic error term. A detailed description of the ordered probit model and the latent variable approach is provided in Winkelmann and Boes as well as Wooldridge [30,31].

## Results

In total, 1,221 students participated in the survey (response rate almost 95%). Due to implausible or missing data, 133 responses were excluded from the regression. Of the 1,088 participants included, 29% are medical students, 33% are law students, 29% are students of economics and 9% study philosophy/religion. In the four groups, the percentage of freshmen is between 60 and 64%. In our study, advanced students have finished 4.6 (±2.2) study terms on average. Among students of economics, 47% are female. In the other fields of study, the proportion is 64–66%.

**Figure 1:**
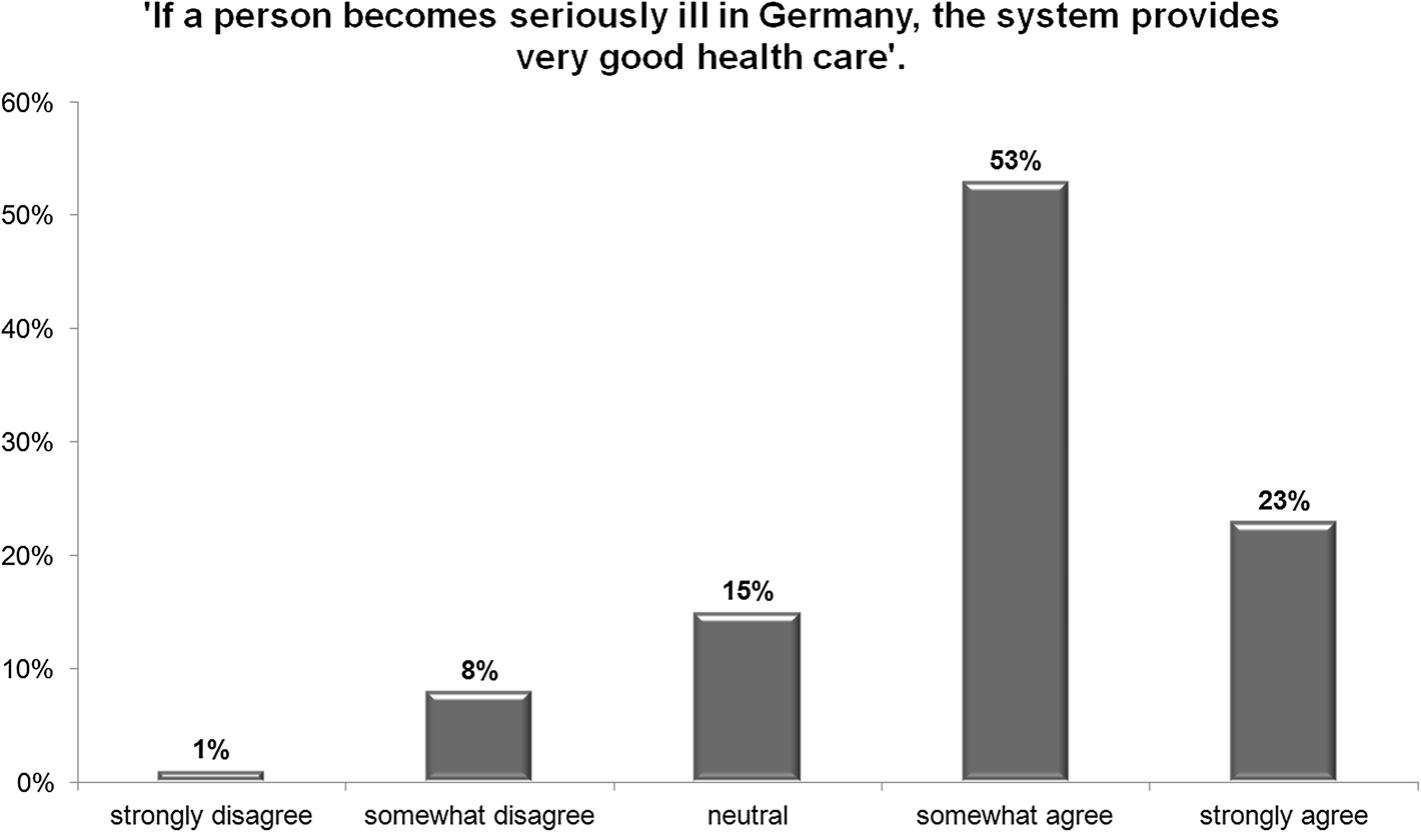
**Overall perception of** ‘**Standard of Care’ (in %).**

**Figure 2:**
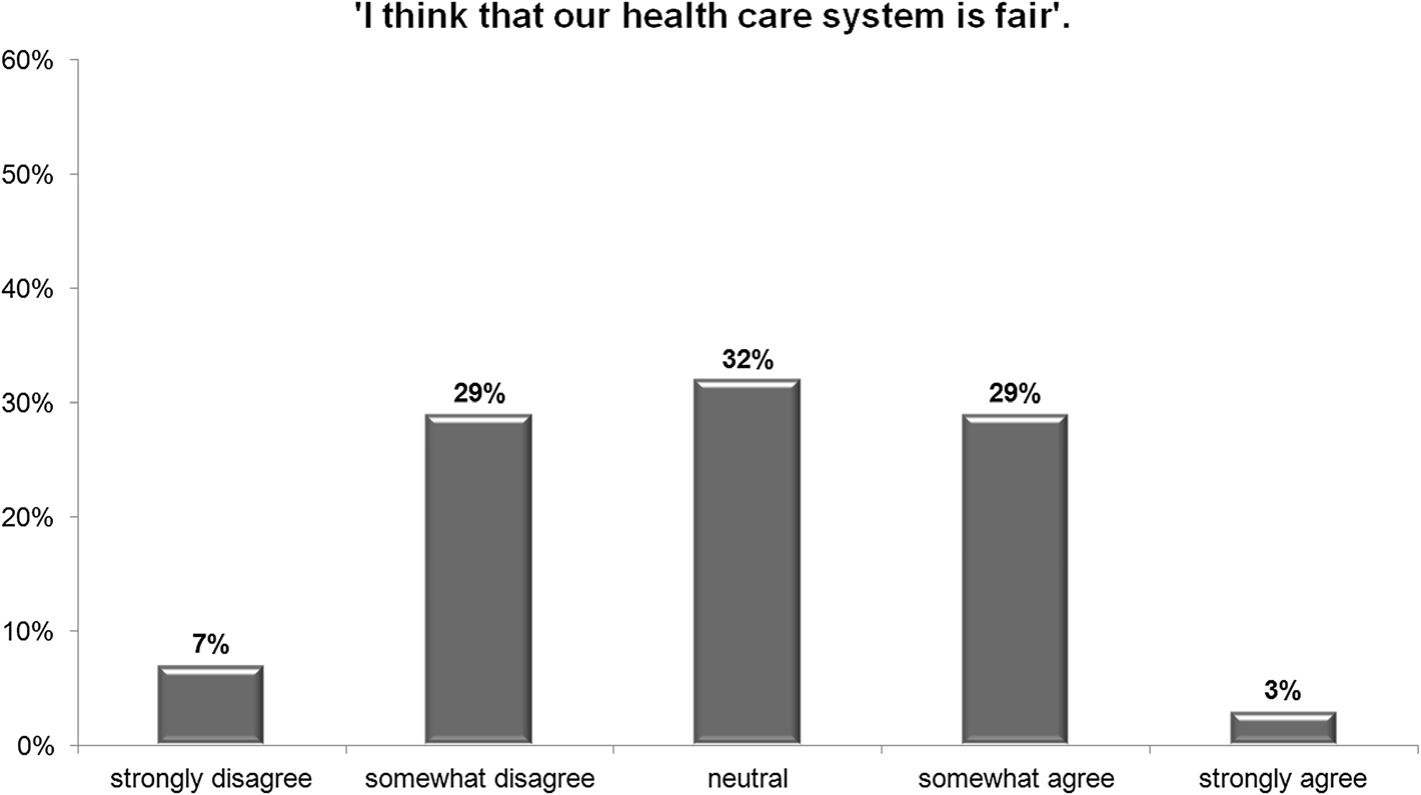
**Overall perception of** ‘**Fairness’ (in %).**

Figures 1 and 2 give a first impression of the responses. Whereas 76% of the participants agreed or somewhat agreed that the standard of care is good, 23% strongly agreed and only 9% somewhat or strongly disagreed. By contrast, 32% somewhat or strongly agreed that the health care system is fair, 32% were neutral, and 36% somewhat or strongly disagreed.

Tables 2 and 3 present the results of the two regressions.

**Table 2:** **Regression results** ‘**Standard of Care**’

**Variable**	**Coefficient (standard error)**
HYPOTHESES RELATED	
Study terms	0.095*** (0.023)
*Academic Discipline* (*Reference*: *Medicine*)	
Law	−0.212* (0.104)
Economics	−0.208* (0.114)
Philosophy/religion	−0.294* (0.151)
*Study terms* × *academic discipline* (*Reference*: *Study terms* × *medicine*)	
Study terms × law	−0.167*** (0.036)
Study terms × economics	−0.063** (0.033)
Study terms × philosophy/religion	−0.098** (0.039)
FURTHER CONTROL VARIABLES	
Gender (*Reference*: *male*)	−0.188*** (0.071)
Type of health insurance (*Reference*: *SHI*)	0.352*** (0.091)
Chronic disease (*Reference*: *no*)	−0.193* (0.099)
*Subj. health status* (*Reference*: *good*)	
Bad	−0.380** (0.179)
Neutral	−0.113 (0.129)
*Subj. health*-*consciousness* (*Reference*: *good*)	
Bad	−0.119 (0.109)
Neutral	−0.234*** (0.089)
Number of observations	1,088
Nagelkerke R^2^	0.111

Insignificant control variables: nationality, children, age, serious illness among family or friends, acute disease, social commitment, parental schooling years.

Significance levels denoted by *:10%, **:5%, ***:1% level [32,33].

SHI: statutory health insurance, Subj.: Subjective.

**Table 3:** **Regression results** ‘**Fairness**’

**Variable**	**Coefficient (standard error)**
HYPOTHESES RELATED	
Study terms	−0.029 (0.032)
*Academic Discipline* (*Reference category*: *Philosophy*/*Religion*)	
Medicine	0.151 (0.146)
Law	0.207 (0.144)
Economics	0.217 (0.150)
*Study terms* × *academic discipline* (*Reference*: *Study terms* × *philosophy*/*religion*)	
Study terms × medicine	0.076** (0.038)
Study terms × law	−0.042 (0.042)
Study terms × economics	0.068* (0.040)
FURTHER CONTROL VARIABLES	
Serious illness among family or friends (*Reference*: *no*)	−0.122* (0.069)
*Subj. health*-*consciousness* (*Reference*: *good*)	
bad	−0.199* (0.106)
Neutral	−0.001 (0.086)
Number of observations	1,088
Nagelkerke R^2^	0.051

Insignificant control variables: gender, nationality, children, age, acute disease, chronic disease, serious illness in family or friends circle, subj. health status, social commitment, parental schooling years.

Significance levels denoted by *:10%, **:5%, ***:1% level [32,33].

Subj.: Subjective.

In the following, we explore the regression results with regard to our hypotheses.

*Hypothesis 1*: *Incoming students perceptions towards standards of care and fairness do not systematically differ between different fields of study*.

With respect to standard of care, Table 2 shows that the coefficients of law, economics, and philosophy/religion are all negative. This indicates that medical students perceive the standard of care as being better than non-medical students at the beginning of their studies^a^. All differences are significant at the 10%-level. Focusing on the size of the coefficients, the difference is largest for law and smallest for economics. A successive change of the reference category does not show any significant difference between law, economics, and philosophy/religion. The first part of Hypothesis 1 therefore cannot be confirmed.

Considering fairness, Table 3 shows that the coefficients of all three academic disciplines are positive. Religion/philosophy students perceive the health care system as less fair compared to students of other disciplines. Unlike in the case of standard of care, the coefficients are not significant. A successive change of the reference category does not produce significant results. The second part of Hypothesis 1 can therefore be confirmed to some extent as no significant difference between religion/philosophy students and the other academic disciplines exists.

*Hypothesis 2*: *Advanced medical students assess the standard of care as being worse compared to the assessments of incoming medical students. In addition*, *advanced medical students view the standard of care as being worse compared to the assessments of advanced students in any other field of study*.

We already demonstrated that medical students perceive the standard of care better compared to all other disciplines. Furthermore, the number of study terms is positive and highly significant (p < 0.01) (Table 2), which indicates that medical students perceive the standard of care increasingly better over the course of their studies. The interaction variables show that advanced law, economics, and philosophy/religion students perceive the standard of care significantly different from advanced medical students, but with negative coefficients. This indicates that the difference in the perception of the standard of care between medical students on the one hand and law, economic, and philosophy/religion students on the other increases with the number of study terms. Combined with the negative coefficient of the other three academic disciplines noted above (see Hypothesis 1), advanced medical students assess the standard of care as being better than advanced students of the other fields of study. Accordingly, our Hypothesis 2 cannot be confirmed.

**Figure 3:**
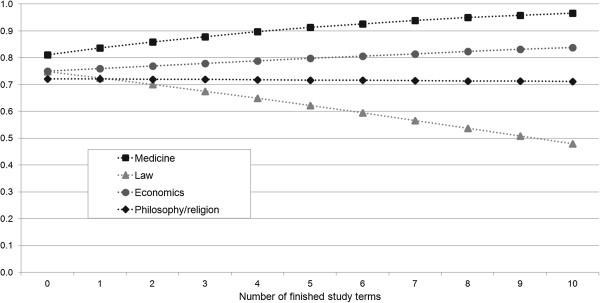
**Predicted probabilities of a reference person to ‘somewhat or strongly agree’ to the question of ‘Standard of Care’.**

To illustrate the effects graphically, Figure 3 shows the predicted probabilities of a reference person to ‘somewhat or strongly agree’ with the statement depending on the number of finished study terms for each field of study. The reference person is characterized by the average values of our continuous variables as well as the highest proportion in the case of the dummy variables (female, SHI, no children, no illness, etc.). The figure demonstrates that the predicted probabilities are higher in the case of medical studies compared to all other fields at the beginning and in each subsequent study term. Furthermore, the gap between medical students and all other fields of study increases as the number of completed study terms increases.

*Hypothesis 3*: *Advanced philosophy*/*religion students assess the fairness of the German health care system as being worse compared to incoming philosophy*/*religion students. Furthermore*, *advanced philosophy*/*religion students view the fairness of the German health care system as being worse compared to the assessments of advanced students in any other field of study*.

This hypothesis can partly be confirmed. The perception of philosophy/religion students decreases only slightly (−0.029). This coefficient is insignificant. The first part of our hypothesis therefore cannot be confirmed.

Freshmen who study philosophy/religion already evaluate the health care system as less fair than students of all the other disciplines, but not significantly (see Hypothesis 1). The interaction terms show that the estimates of the economics and medical students are significantly different to the reference category, with both coefficients being positive. As a result, the difference in perception between philosophy/religion students on the one hand and economics and medical students on the other increases with the number of study terms. Thus, advanced students of philosophy/religion assess the fairness of the system worse compared to medical and economics students. Accordingly, the second part of this hypothesis can be confirmed in the cases of medical and economic students.

With respect to the perception of advanced law students, the interaction term is negative but insignificant. Based on the initial non-significant positive coefficient of law students, the difference between philosophy/religion and law students is even diminishing with the number of study terms as the perception of the latter decreases on a higher extent (−0.071, p < 0.05). Accordingly, it cannot be confirmed that advanced students of philosophy/religion assess the fairness in the German health care system in general worse compared to the assessments of advanced law students.

**Figure 4:**
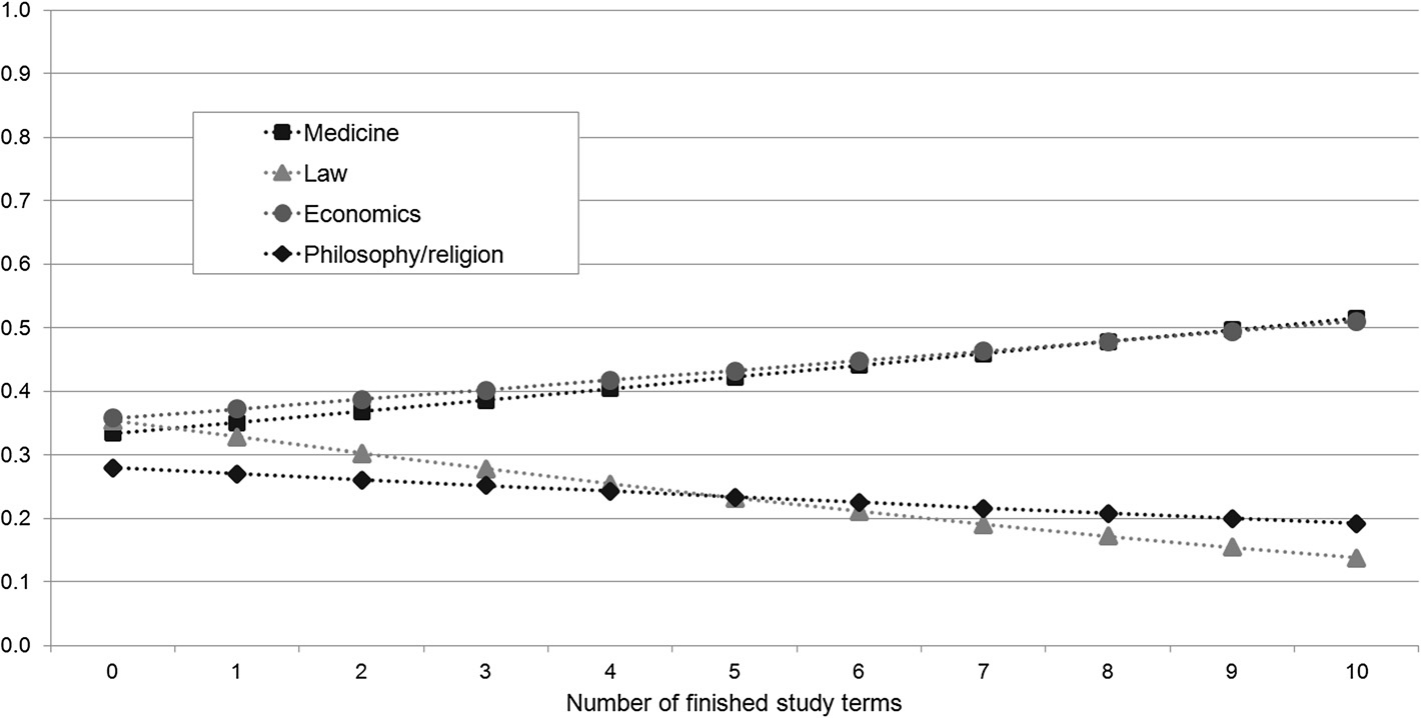
**Predicted probabilities of a reference person to ‘somewhat or strongly agree’ to the question of ‘Fairness’.**

These results are also illustrated in Figure 4. The reference person has the same characteristics as in Figure 3. As an example, we show the predicted probabilities of our reference person to respond to the statement about fairness with ‘somewhat or strongly agree’, depending on the number of finished study terms for each field of study. The figure illustrates that in the case of philosophy/religion studies our reference person is already less likely to agree with the statement about fairness as a freshman. In each subsequent study term, the predicted probability decreases, but to a lesser extent than in case of law studies, as noted above. The intersection point of both curves is after the fifth study term.

Independently from the hypotheses, Table 2 indicates that other variables have a significant impact on the assessment of health care. The coefficient for gender has a highly significant influence (p < 0.01). Female students rate the standard of care as being worse in comparison to male students. The coefficient for type of health insurance is also highly significant. Students who are covered by PHI estimate the standard of care as being better than students covered by SHI. Participants with a chronic illness rate the standard of care as being worse. In addition, people with a worse subjective health status have a worse perception of the standard of care. This also applies to the value of the coefficient ‘health-conscious lifestyle’: a higher subjective perception of individual lifestyle coincides with a perception of the standard of care as being better.

Table 3 shows that a serious illness among family or friends has a significant negative impact on the perception of fairness. Again, the coefficients of the variables for ‘health-conscious lifestyle’ are negative but only significant for the category ‘bad’ at the 10%-level.

## Discussion

The analyses show that the particular field of study has an impact on the perception of standard of care and fairness in the health care system. This finding is in line with Ahlert, Schwettmann et al. and Ahlert, Felder et al., who conducted experiments with students on the distribution of resources in an equitable health system [34,35]. The authors also found differences in behaviour between academic disciplines. With respect to standard of care, we showed that – contrary to our hypothesis – medical students begin their studies more optimistic than students in non-medical disciplines. Their perception increases, moreover, over the course of their studies. Zupanic et al. asked medical students about their motivations to study medicine [21]. The main aspects were an interest in medical issues, contact with people as well as desire to help. At first sight, it seems that there is no direct connection between the motivations and their perception of standard of care, but medical freshmen may already be convinced of the system; otherwise, they might have been deterred from studying medicine in the first place. On the one hand, the growing optimism over the course of their studies may contradict some of the results found by Osenberg et al. and Zupanic et al. [21,22]. Their studies show that German medical students anticipate the budgeting of services as an important problem of their future work, perceive deficiencies in patient care, and are concerned about those in the future. It can also be assumed that medical students perceive the problem of resource allocation in more direct and specific terms [36]. Only recently, the professional association of medical students in Germany requested an open debate about rationing [37]. On the other hand, there are arguments that might explain the growing optimism. Medical students are taught by professors at medical universities who do basic research and take care of serious conditions. In general, this basic research intends to deliver innovative therapies for the treatment of patients. This environment might also influence the students’ perception. It can also be assumed that medical students learn about the health systems of other countries in ways unlike non-medical students. It may be that their responses have a more relative character. An international survey conducted by Koch et al. showed that German practitioners require comprehensive reforms of the health care system but overall assess the standard of care as being good [38].

In contrast to the question regarding the standard of care, there was no significant difference between the freshmen`s perception of fairness, but the coefficients indicate that philosophy/religion students make up the most critical of all groups. The non-significance might result from the relatively small size of the sample.

More interesting, however, is that there is only a marginal difference between the perception of freshmen and advanced students studying philosophy/religion. Here, our assumption that the course of the philosophy/religion studies influences the perception of fairness in the health care system cannot be confirmed. Instead, our results show that the law students’ perception of fairness decreases the most. Considered individually, the perception of the law students decreases significantly (p < 0.05) during the course of study. Of course, it may be that philosophy/religion freshmen already have a relatively well-grounded, consolidated opinion. In comparison to other freshmen, the perception of philosophy/religion students does not significantly differ (see Table 3). Looking at the coefficients, however, their perception is already worse, which might explain the marginal changes during the course of study. Another reason might be that the health care system is not discussed directly in the courses of philosophy/religion students. The coefficients nevertheless show that the perception of advanced philosophy/religion students is worse compared to advanced medical and economics students. Additionally, Figure 4 illustrates how the perceptions of law and philosophy/religion students and economics and medicine students diverge. Studies have shown that medical school can often have detrimental effects like increasing cynicism and decreasing empathy on students' professional growth [39-41]. If these aspects played a role here remains open. The results however may indicate a high conflict potential between the disciplines and may also have an impact on the controversial discussion over health care reforms, prioritization and rationing in the health care sector [19,20].

Independently of the hypotheses, we generated additional results. The type of health insurance and gender have a high significant influence on the assessment of standard of care. In our study population, men and persons covered by PHI rate the standard of care as better than persons covered by SHI. These findings are in line with those of Sawicki, who obtained similar results regarding the standard of care [42]. In a patient survey, he found out that the insurance type has a relevant influence on the assessment and that women have a worse perception of the standard of care. A citizen survey of the European Commission analysed the socio-demographic factors that determine a poor subjective assessment of the quality of health care [43]. No differences between men and women were found, but the authors recoded the variable ‘perceptions’ in only two categories (good/bad), which might have led to a loss of information. Furthermore, the question stressed the quality of health care, which is not exactly the focus of our inquiry.

In addition, our analysis revealed that subjective health status has an impact on the perception of the standard of care. Here, the coefficients for chronic disease, poor subjective health status, and less health-conscious lifestyle are all negative. It can be assumed that people with chronic disease have more experience with the health care system. In an international study by Schoen et al., German patients criticized deficiencies in care and poor coordination for chronic conditions in Germany [44,45]. The study also revealed that German patients had a more negative perception compared to foreign subjects, but the negative impact that we discern also contradicts the results of previous studies. Zuchandke et al. and Lange et al., who investigated the perception of financial security in cases of long-term care need and illness, determined that people with experience of the German health care system and long-term care perceive their financial security better compared to those without such experience [46,47]. One could therefore assume that this would also be the case for standard of care, but these perceptions are of course not directly comparable.

Unlike the first regression, fewer control variables have a significant impact on the perception of fairness in the health care system. In particular, there is no significant difference between women and men. As in the first regression, participants with a less subjectively health-conscious lifestyle assess the system as less fair. This is interesting, since the provision of health care in Germany should not be dependent on the lifestyle. Another interesting result is that the variable ‘serious illness among family or friends’ has a significant negative impact on the assessment of fairness. About 33% of the study participants report having at least one person with serious illness among family or friends, but they do not assess the standard of care as significantly worse. Why the perception of fairness is more negative in this group raises a new research question.

In what follows, we highlight limitations with respect to the variables used, the methodology, and the study population. The objective of our study was to determine whether the assessment of the German health care system in terms of standard of care and fairness differs between fields of study. We used two statements as proxies. Although we can describe certain tendencies in the responses, we cannot indicate, for example, which attributes make up a fair system for the participants. In particular, we do not know whether the students assess the standard of care and fairness of the system in comparison to other systems or with respect to an idea of an optimal system. Additionally, we do not know the participants' actual knowledge of the health care system and to what extent a combination of knowledge and views affect each other. However, we found a significant correlation (p < 0.01) between both variables (standard of care and fairness). Respondents might somehow have experienced a form of rationing that underpinned both an unfavourable assessment of standard of care and of fairness.

In this context, we want to stress the subjective character of our dependent variables, which can lead to heterogeneous interpretations. Unfortunately, we are not able to control for such interpersonal heterogeneity as we do not have panel data. While the heterogeneity can be reduced by transforming the ordinal variables to binary variables, this would lead to a loss of information and consequently less precise results. We therefore decided to keep the ordinal structure of our dependent variables. However, further research is needed to identify the ideas of an optimal system in terms of standard of care and fairness. A mixed methods approach consisting of combined quantitative and qualitative surveys might be appropriate. Additionally, further questions regarding a fair priority setting could be addressed here, including the identification of relevant attributes, the balancing of values like efficiency, equity, or reasonableness, and trade-off between different focuses (achieving fair outcomes or a fair process).

With respect to the dependent variable ‘standard of care’, we want to highlight that our statement focused on the standard of care of seriously ill patients. The general label ‘standard of care’ may therefore be misleading. We nevertheless decided to formulate the statement more precisely since previous studies showed that the health care of seriously ill patients with high severity is more important to society than the health care of minor conditions [48,49].

A problem of imperfect multicollinearity may result due to the inclusion of ‘year of birth’ as a control variable. There is obviously a high correlation between age and the number of study terms (p < 0.01). We nevertheless wanted to control for possible age differences among freshmen, but we have also redone the regression analyses without considering the factor ‘age’ to test the impact on the standard errors. The significance levels did not change.

Ordered probit and ordered logit models are often used in applied econometric analyses. The models nevertheless have some limitations especially when it comes to analysing marginal effects. First of all, the assumption of a normal or logistic distribution leads to the case in which the sign of the coefficients does not necessarily represent the direction of the effect for all outcomes of the dependent ordinal variable [31]. Moreover, assumptions such as the single index function or constant thresholds lead to further restrictions. A detailed overview of limitations based on ordered response models is provided in Boes and Winkelmann [50].

With respect to the subjects, they all studied in Hannover or Göttingen. It is possible that students in southern or eastern Germany have a different perception. It is also possible that advanced students have been influenced by the opinion of single professors at these universities. Since lectures are given by various professors, however, the possibility of a special influence is minor. A wider study would nevertheless also have the advantage that more students who study philosophy and religion could be integrated into the data set. Due to the small number of students, we formed one group from these two subjects, which is also a restriction. We therefore implicitly assume both groups to be identical with regard to the perception of ‘standard of care’ and ‘fairness’.

As already mentioned above, we used cross-sectional data instead of longitudinal data. We did not analyse the perception of the same study group over time. Besides the mentioned possibility of controlling for interpersonal heterogeneity, another important issue is the dropout rate. This may especially be relevant for law and economics, in which the rate is up to 50%. It would therefore be interesting to conduct the same study again and interrogate the same group over time.

Finally, we want to stress that the results of this study are not representative for the general population, since students are young and well educated. Age and the socio-economic status may have an important influence on the perception of fairness and standard of care. It would therefore be interesting to repeat the study, ask the general population about their opinion, and control for the age and socio-economic status of the subjects.

## Conclusions

To our knowledge, this is the first study to examine the influence of the field of study on the perception of fairness and standard of care in the German health care system via extensive statistical analyses. The results give a possible explanation for the challenging interdisciplinary debates on health care financing and service provision. Physicians might struggle with rationing instruments in the German health care system due to their good perception of the health care system. It may be harder for them to accept further cuts in the statutory benefit catalogue or fewer approvals and reimbursements for new devises and therapies. Such restrictions may endanger their highly perceived health care standard. The differences in the perception of fairness are not as obvious as we expected. The results nevertheless show that differences exist, which might influence interdisciplinary discussions on health care reforms, prioritization and rationing. Our study, however, focuses only on subjective perception. Further research is necessary to determine the attributes that make up a high standard of care and fair system.

## Endnote

^a^To avoid misunderstandings, we would like to emphasize that we refer the term “better perception” or “perceive better” in sense of a more favorable perception and not as a more accurate perception throughout the whole paper.

## Competing interests

The authors declare no conflict of interest.

## Authors’ contributions

KD and AP designed and coordinated the study and drafted the manuscript. KD and AZ performed the statistical analysis. AZ helped to draft the manuscript. All authors read and approved the final manuscript.

## Pre-publication history

The pre-publication history for this paper can be accessed here:

http://www.biomedcentral.com/1472-6963/14/166/prepub
